# Retraction: MicroRNA-137 Contributes to Dampened Tumorigenesis in Human Gastric Cancer by Targeting AKT2

**DOI:** 10.1371/journal.pone.0282977

**Published:** 2023-03-29

**Authors:** The PLOS ONE Editors

The *PLOS ONE* Editors retract this article [1] because it was identified as one of a series of submissions for which we have concerns about peer review, authorship, and similarities across articles. Image similarities were noted between Fig 2c in [1] and Fig 2C in [2], and Fig 3b in [1] and Fig and 3B in [2], between Fig 2E in [1] and Fig 6E in [3], and between Fig 5C in [1] and Fig 2D in [4, 5]. These concerns call into question the validity and provenance of the reported results. We regret that the issues were not addressed prior to the article’s publication.

All authors either did not respond directly or could not be reached.
